# LIF/LIFR oncogenic signaling is a novel therapeutic target in endometrial cancer

**DOI:** 10.1038/s41420-021-00603-z

**Published:** 2021-08-16

**Authors:** Weiwei Tang, Kumaraguruparan Ramasamy, Sureshkumar M. A. Pillai, Bindu Santhamma, Swapna Konda, Prabhakar Pitta Venkata, Logan Blankenship, Junhao Liu, Zexuan Liu, Kristin A. Altwegg, Behnam Ebrahimi, Uday P. Pratap, Xiaonan Li, Philip T. Valente, Edward Kost, Gangadhara R. Sareddy, Ratna K. Vadlamudi, Hareesh B. Nair, Rajeshwar R. Tekmal, Suryavathi Viswanadhapalli

**Affiliations:** 1 Department of Obstetrics and Gynecology, University of Texas Health San Antonio, San Antonio, TX 78229 USA; 2 Department of Obstetrics and Gynecology, Affiliated Hospital of Integrated Traditional Chinese and Western Medicine, Nanjing University of Chinese Medicine, 210028 Nanjing, China; 3 Evestra, Inc., San Antonio, TX 78245 USA; 4 Department of Oncology, Xiangya Hospital, Central South University, 410008 Hunan, China; 5 Mays Cancer Center, University of Texas Health San Antonio, San Antonio, TX 78229 USA

**Keywords:** Endometrial cancer, Target validation

## Abstract

Endometrial cancer (EC) is the fourth most common cancer in women. Advanced-stage EC has limited treatment options with a poor prognosis. There is an unmet need for the identification of actionable drivers for the development of targeted therapies in EC. Leukemia inhibitory factor receptor (LIFR) and its ligand LIF play a major role in cancer progression, metastasis, stemness, and therapy resistance. However, little is known about the functional significance of the LIF/LIFR axis in EC progression. In this study using endometrial tumor tissue arrays, we identified that expression of LIF, LIFR is upregulated in EC. Knockout of LIFR using CRISPR/Cas9 in two different EC cells resulted in a significant reduction of their cell viability and cell survival. In vivo studies demonstrated that LIFR-KO significantly reduced EC xenograft tumor growth. Treatment of established and primary patient-derived EC cells with a novel LIFR inhibitor, EC359 resulted in the reduction of cell viability with an IC_50_ in the range of 20–100 nM and induction of apoptosis. Further, treatment with EC359 reduced the spheroid formation of EC cancer stem cells and reduced the levels of cancer stem cell markers SOX2, OCT4, NANOG, and Axin2. Mechanistic studies demonstrated that EC359 treatment attenuated the activation of LIF-LIFR driven pathways, including STAT3 and AKT/mTOR signaling in EC cells. Importantly, EC359 treatment resulted in a significant reduction of the growth of EC patient-derived explants ex vivo, EC cell line-derived xenografts, and patient-derived xenografts in vivo. Collectively, our work revealed the oncogenic potential of the LIF/LIFR axis in EC and support the utility of LIFR inhibitor, EC359, as a novel targeted therapy for EC via the inhibition of LIF/LIFR oncogenic signaling.

## Introduction

Endometrial cancer (EC) accounts for ~76,000 deaths among women worldwide each year. It is the sixth leading cause of cancer death among women in the United States [1]. EC is comprised of four histological subtypes, including endometrioid endometrial cancer (EEC), serous endometrial cancer (SEC), clear cell endometrial cancer (CCEC), mixed EC and uterine carcinosarcoma [1]. Approximately 80% of EC cases belong to the endometrioid subtype and are driven by estrogen (E2, 17-β-estradiol) signaling [2]. Imbalance between E2 and progesterone exposures and the use of unopposed E2 therapy are implicated as major risk factors for EC [3]. Despite the promising results of progestins for patients receiving uterine-sparing treatment, adjuvant hormone therapy has not been shown to confer benefit to EC patients after surgery [4] and the recurrence rate is ~50%. Advanced-stage EC has limited treatment options and poor prognosis [5]. A better understanding of the molecular drivers of EC progression is needed to develop effective targeted therapies for EC.

Leukemia inhibitory factor (LIF) is the most pleiotropic member of the interleukin-6 family of cytokines [6]. LIF signaling is mediated via the LIF receptor (LIFR) complex that is comprised of LIFR and glycoprotein 130 (gp130) [7]. LIF activates multiple signaling pathways via its interaction with LIFR including STAT3, AKT, MAPK, and mTOR [7–9]. Interestingly, LIFR does not have intrinsic tyrosine kinase activity. Both the LIFR and gp130 constitutively associate with the JAK-Tyk family of cytoplasmic tyrosine kinases. Therefore, when LIF binds to the LIFR complex it leads to activation of the JAK/STAT pathway [7]. The LIF/LIFR axis is implicated in tumor growth and progression by altering the magnitude of several oncogenic processes [10, 11]. In addition, the LIF/LIFR axis is also implicated in the maintenance of stem cells [12, 13], and the deregulation of LIF/LIFR signaling contributes to chemoresistance [14, 15].

EC is estimated to increase by 1–2% yearly and the obesity epidemic further implicates an increase of EC cases in the USA [16] and is an independent risk factor for EC [17]. Cellular components of adipose tissue are the predominant source of aromatase, the enzyme that facilitates the production of estrogen [18]. Obesity signals (such as estrogen, leptin) promote EC [19] and function as potent inducers of LIF [20, 21]. LIF is an established E2-responsive gene in the uterus [22]. Obese people have unusually high levels of leptin; and leptin increases p-STAT3, LIF, LIFR, levels in cultured human endometrial cells [21]. LIF is a commonly upregulated gene in carboplatin- and paclitaxel-resistant cells and its expression correlates with poor outcome in EC patients [23]. Collectively, these findings suggest that LIF/LIFR may function as a novel therapeutic target for EC, however, its role and mechanisms in the progression of EC remain elusive.

In this study, we have examined the role of LIF/LIFR signaling in EC progression. A global analysis of EC gene expression databases revealed the negative correlation between EC survival and the expression of both LIF and LIFR. Using LIFR Knockout (KO) model cell lines, we provide genetic evidence that intrinsic LIF/LIFR signaling will benefit EC progression. Our work has shown that novel LIFR inhibitor, EC359, reduces the growth of EC cell lines with high potency and promotes apoptosis. Preclinical xenograft, patient-derived explant, and xenograft studies demonstrated that LIFR inhibitor, EC359 is potent in reducing EC tumor growth and the therapy is well tolerated in vivo.

## Results

### EC tissues express higher levels of LIF and LIFR

**Fig. 1:**
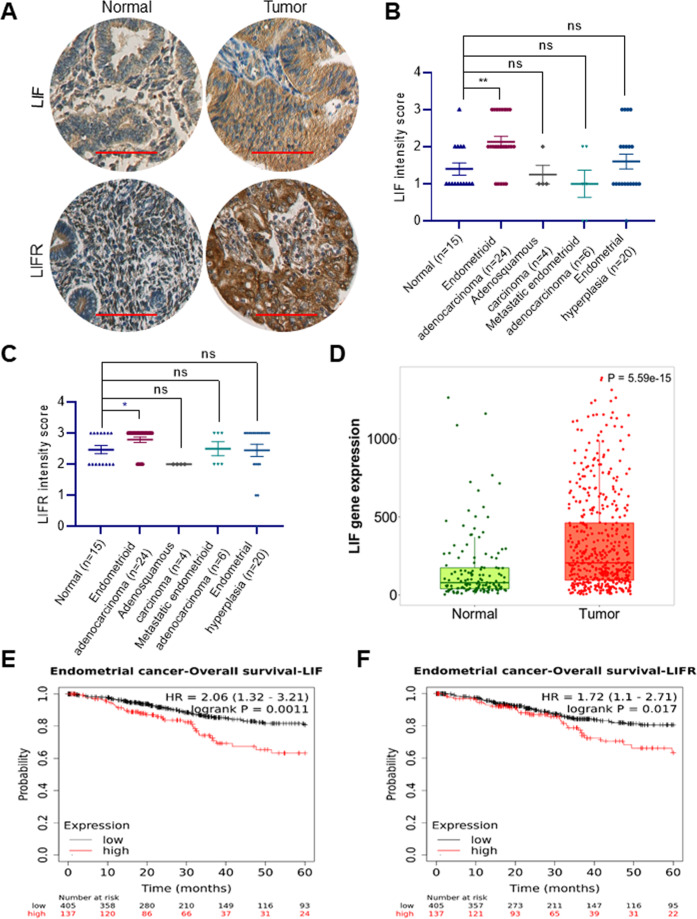
LIF and LIFR are overexpressed in EC. **A** Representative IHC images of LIF and LIFR expression in normal and EC. Scale bar represents 100 µm. **B**, **C** Quantitation of expression of LIF and LIFR in normal (*n* = 15) and different subtypes of EC (*n* = 54) from commercial TMA. **D** Boxplots of LIF gene expression in normal (*n* = 146) and EC (*n* = 547) gene array data. **E**, **F** Association of LIF and LIFR expression with survival of EC patients was analyzed using Kaplan–Meier survival analysis tool (KMplot). Data are represented as mean ± SE. *p < 0.05, **p < 0.01, ns not significant.

We examined the expression of LIF and LIFR immunohistochemically using commercial TMA that contained 15 normal and 54 EC specimens. The intensity of staining and positivity was recorded by the pathologist. The representative staining in EC and normal tissue is shown in Fig. 1A. IHC analysis revealed that the expression of LIF and LIFR was significantly greater in the endometrioid adenocarcinoma subtype compared to normal tissues (Fig. 1B, C). We also examined LIF status in EC using publicly available TNMplot analysis platform that enable comparison of gene expression between tumor and normal tissues using validated database [24]. Results showed that LIF is highly expressed in EC compared to normal tissues (Fig. 1D). Analyses of the association of LIF and LIFR expression with survival of EC patients using the Kaplan–Meier survival analysis tool (https://kmplot.com/analysis/) showed increased LIF and LIFR expression was associated with poor overall survival (OS) (Fig. 1E, F). These results confirmed that LIF/LIFR axis is increased in EC and may be associated with poor OS.

### Functional LIF/LIFR is needed for optimal growth of EC in vivo

**Fig. 2:**
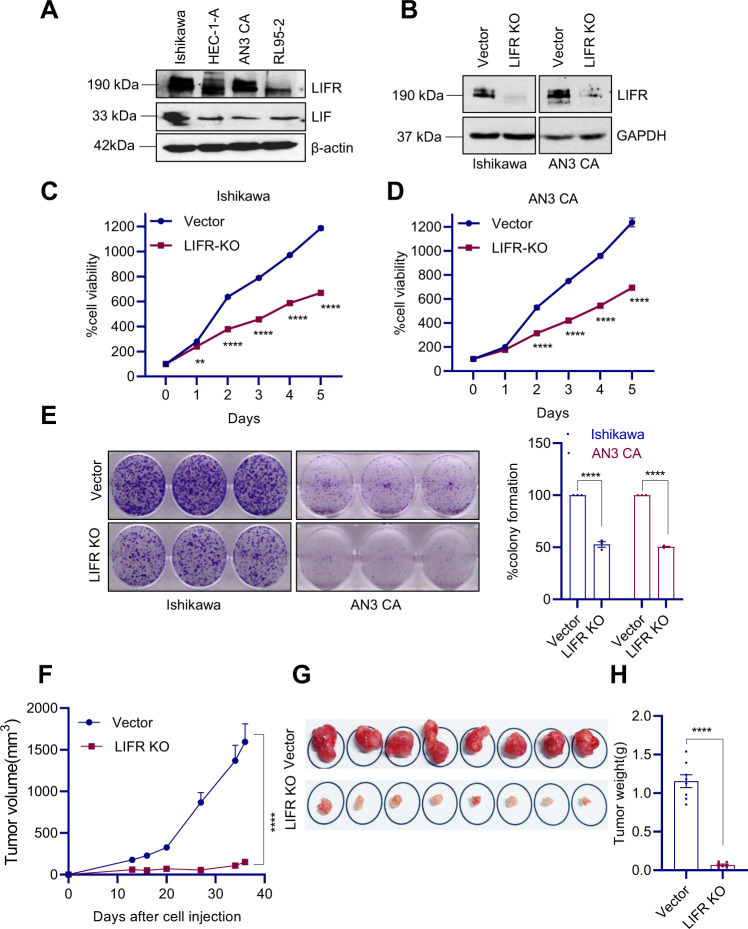
Knockout of LIFR reduced EC progression in vivo. **A** Expression of LIF and LIFR in EC cells was analyzed by western blotting. **B** Levels of LIFR in CRISPR/Cas9-mediated KO Ishikawa and AN3 CA cells were measured by western blotting. The effect of LIFR-KO on cell viability in Ishikawa (**C**) and AN3 CA (**D**) cells was measured using MTT assays. **E** Effect of LIFR-KO on Ishikawa and AN3 CA cell survival was measured using colony formation assays. Quantitation was shown in the right panel. **F** Ishikawa-vector or Ishikawa-LIFR-KO cells were injected subcutaneously into SCID mice (*n* = 8) and tumor growth was monitored. Tumor pictures (**G**) and tumor weights (**H**) were shown. Data are represented as mean ± SE. **p < 0.01, ****p < 0.0001.

We initially examined the expression of LIF and LIFR in widely used EC model cell lines. Western blotting results confirmed that all the four established EC cell lines express both LIF and LIFR (Fig. 2A). To provide genetic evidence that intrinsic LIF/LIFR signaling in EC cells was beneficial to EC progression, we generated Ishikawa and AN3 CA LIFR Knockout (KO) cells using the CRISPR/Cas9 system. Western blot analyses confirmed KO of LIFR (Fig. 2B). Cell viability and colony formation assays demonstrated the reduced growth of Ishikawa and AN3 CA LIFR-KO cells compared to vector controls (Fig. 2C–E). We next determined whether the LIFR-KO could reduce tumor progression in vivo using a mice xenograft model. As shown in Fig. 2F, G, LIFR-KO significantly reduced EC xenograft tumor growth compared to vector controls. Further, the tumor weights are significantly lower in LIFR-KO groups compared to the vector control group (Fig. 2H). These results suggested that LIFR is essential for EC progression in vitro and in vivo.

### LIFR inhibitor EC359 inhibited the cell viability, survival, and promoted the apoptosis of EC cells

**Fig. 3:**
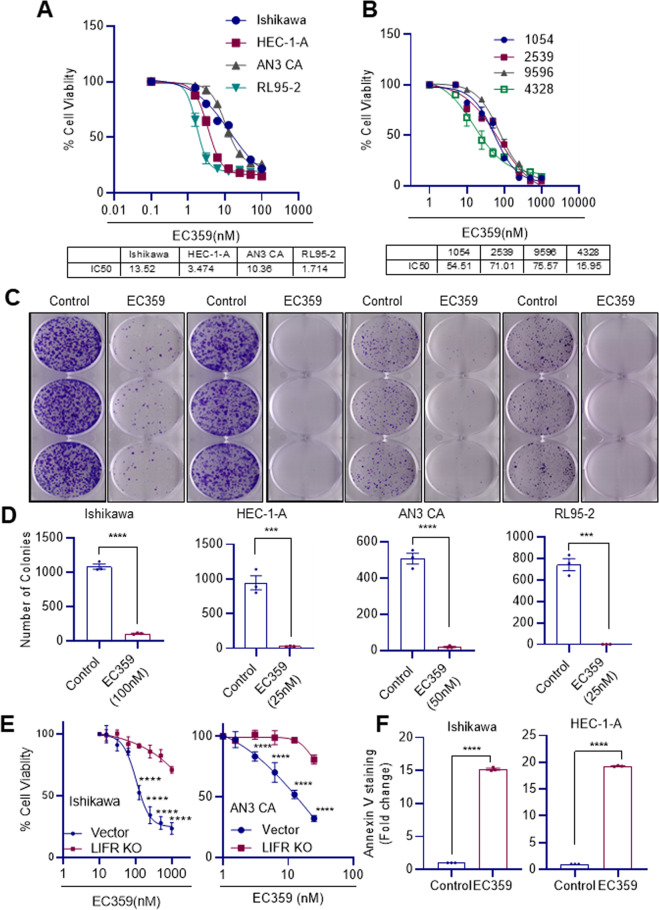
LIFR inhibitor EC359 reduced cell viability, colony formation and promoted apoptosis of EC cells. Effect of EC359 on cell viability of established and patient-derived primary EC (**A**, **B**) cells was determined using MTT assay. **C** Effect of EC359 on cell survival of EC cells was measured using colony formation assays. **D** Quantitation of the number of colonies is shown. **E** Effect of CRISPR/Cas9-mediated KO of LIFR on EC359-induced cell viability was determined using MTT assays in Ishikawa and AN3 CA cells. **F** Effect of EC359 (200 nM) on apoptosis of Ishikawa and HEC-1-A cells (*n* = 3) was determined using Annexin V staining. Data are represented as mean ± SE. ***p < 0.001, ****p < 0.0001.

We recently developed a small molecule inhibitor that specifically targets the LIFR using a rationalized design based on the crystal structure of LIF-LIFR [25]. We tested the biological activity of EC359 on cell viability of established EC cell lines (Ishikawa, HEC-1-A, AN3 CA, RL95-2) and patient-derived primary EC cells (4328, 1054, 2539, 9596) using MTT assay. Results demonstrated that EC359 shows an IC_50_ range of 20–100 nM, suggesting the potent inhibitory activity of EC359 in reducing the cell viability of EC cells (Fig. 3A, B). Furthermore, colony formation assays demonstrated that EC359 significantly reduces the survival of EC cells (Fig. 3C, D). Importantly the specificity of the EC359 was validated using LIFR-KO cells. As shown in Fig. 3E, LIFR-KO in both Ishikawa and AN3 CA compromised the activity of EC359 to reduce the viability of both cells. To test whether EC359 promotes apoptosis, Ishikawa and HEC-1-A cells were treated with EC359 and the apoptosis was measured using Annexin V/PI staining assay. Results showed that EC359 treatment induces apoptosis (Fig. 3F). Altogether, these findings indicated that EC359 specifically inhibits LIFR and is highly potent in reducing cell viability, survival, and inducing apoptosis in EC cells.

### EC359 reduced STAT3 reporter activity and STAT3 target gene expression

**Fig. 4:**
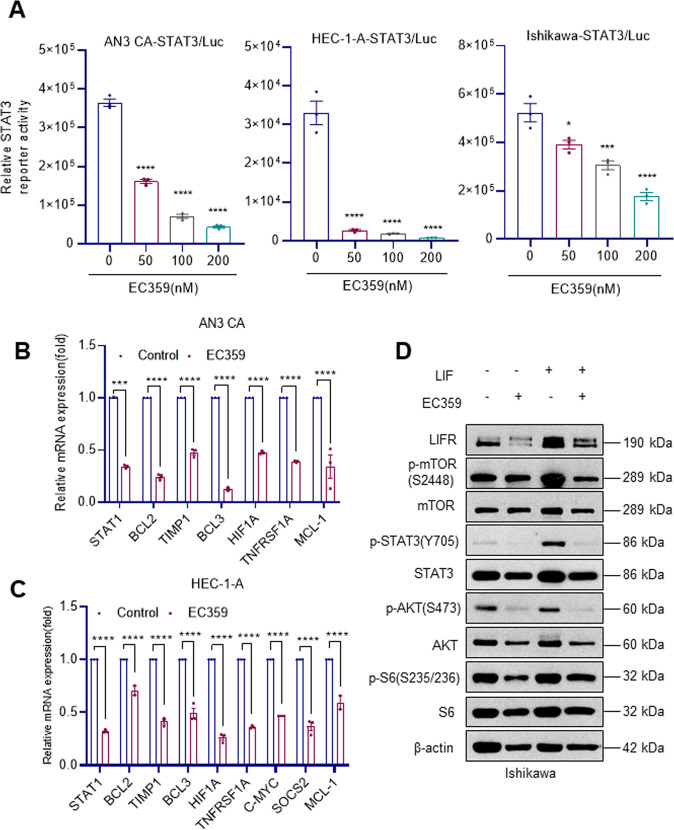
Effect of EC359 on LIFR signaling in EC cells. **A** EC cells stably expressing STAT3-luc reporter were treated with the indicated concentration of EC359. Reporter activity was measured after 24 h. **B**, **C** Effect of EC359 (200 nM) treatment (24 h) on STAT3 target genes was measured using RT-qPCR analysis (*n* = 3). **D** EC cells are pretreated with EC359 for 24 h followed by stimulation with LIF for 30 min and the status of STAT3 signaling was measured by western blotting. Data are represented as mean ± SE. *p < 0.05, **p < 0.01, ***p < 0.001, ****p < 0.0001.

LIF/LIFR activation enhances the activity of STAT3 as an immediate effector. To confirm the inhibitory effect of EC359 on LIF/LIFR-mediated STAT3 activation, AN3 CA, Ishikawa, and HEC-1-A cells that stably express the STAT3-Luc reporter were treated with vehicle or EC359. Treatment with EC359 significantly decreased the STAT3 reporter activity suggesting the existence of LIF/LIFR autocrine loop in EC cells (Fig. 4A). Accordingly, treatment of EC cells with EC359 significantly reduced the expression of STAT3 target genes (Fig. 4B, C). Further, western blot analysis revealed that EC359 treatment substantially reduced the activation of LIFR downstream signaling molecules such as STAT3, Akt, mTOR, and pS6 in Ishikawa cells (Fig. 4D) suggesting that EC359-mediated inhibitory activities on EC cells involves downregulation of STAT3 and mTOR signaling.

### EC359 reduced the viability and self-renewal of cancer stem cells

**Fig. 5:**
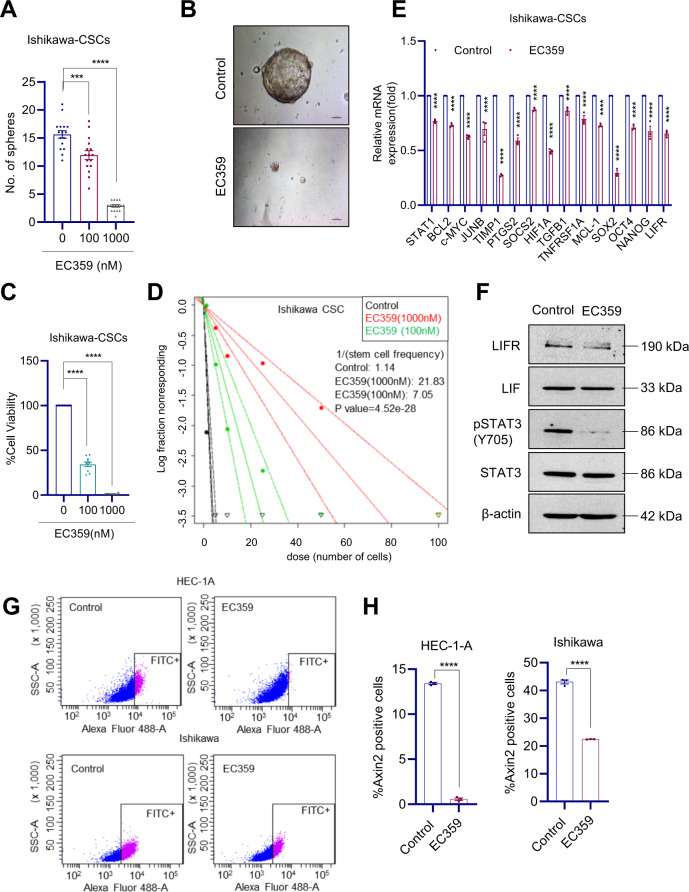
EC359 reduced stemness of EC cells. **A** Sphere formation assay was analyzed by seeding an equal number (100 cells/well) of CSCs and counted the number of spheres after 7 days of vehicle or EC359 treatment. **B** Representative images of Ishikawa-CSCs treated with either vehicle or EC359 (1000 nM) were shown. **C** The effect of EC359 on the viability of CSCs was determined using CellTiter-Glo assay (*n* = 3). **D** Ishikawa-CSCs were seeded in decreasing numbers (100, 50, 20, 10, 5, and 1 cell/well) in 96-well plates and treated with vehicle or EC359, and the number of wells containing spheres per each plating density was recorded after 14 days. The stem-cell frequency between control and treatment groups was calculated using ELDA software. **E** Effect of EC359 on STAT3 target and stemness genes was measured using RT-qPCR analysis. **F** Effect of EC359 on STAT3 signaling was measured by western blotting. HEC-1A and Ishikawa cells were treated with EC359 (200 nM) and after 48 h, the proportion of Axin2-positive cells were determined using flow cytometry (**G**) and quantitation is shown (**H**). Data are represented as mean ± SE. ***p < 0.001, ****p < 0.0001.

The LIF/LIFR axis plays a vital role in stemness [12, 26]. Therefore, to test the effect of EC359 on stemness, CSCs were isolated from Ishikawa cells using ALDH^+^ sorting with flow cytometry. EC359 treatment of CSCs reduced the number of spheroids (Fig. 5A, B) and inhibited cell viability (Fig. 5C). Further, EC359 treatment resulted in decreased self-renewal ability of CSCs compared to control (Fig. 5D). To examine whether EC359 treatment reduces STAT3 signaling in CSCs, RT-qPCR and western blot analyses were performed. Results showed that EC359 treatment significantly reduced the expression of STAT3 target genes as well as stemness genes including SOX2, OCT4, and NANOG (Fig. 5E) and substantially decreased the phosphorylation of STAT3 (Fig. 5F). Recent studies identified Axin2, a classical Wnt reporter gene as a robust stem-cell marker in endometrial cancer [27]. We then examined the effect of EC359 on Axin2-positive CSC populations using flow cytometry. Treatment of HEC-1-A and Ishikawa cells with EC359 resulted in a significant reduction of Axin2-positive CSCs compared to controls (Fig. 5G, H). Collectively, these results suggest that EC359 is efficacious in reducing the stemness of CSCs.

### EC359 suppressed EC xenograft tumor growth in vivo

**Fig. 6:**
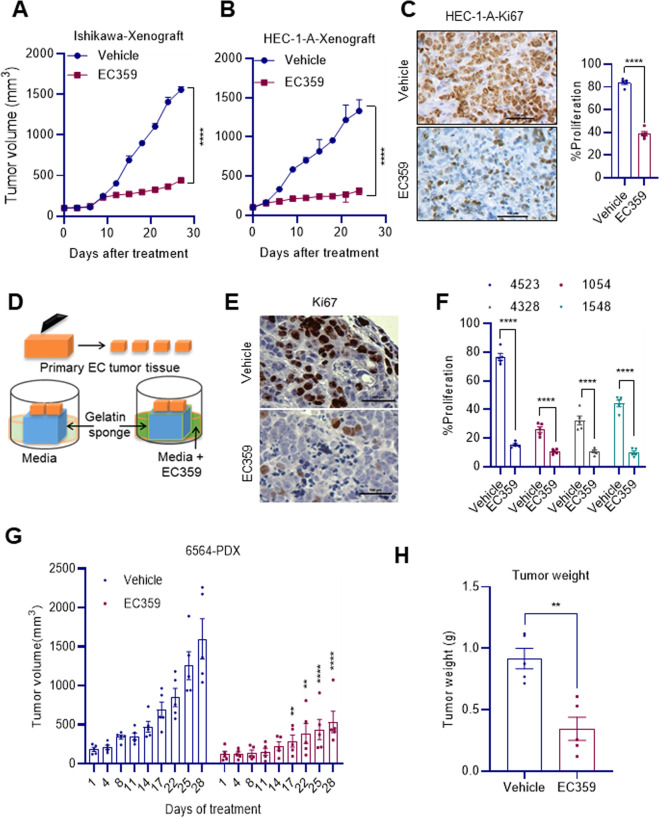
EC359 inhibited the growth of EC xenograft, PDEx, and PDX tumors. Ishikawa (**A**) and HEC-1-A (**B**) xenograft tumors were treated with vehicle or EC359 (2.5 mg/kg/day/s.c/6 days/week). Tumor volume is measured twice a week. **C** Vehicle and EC359-treated HEC-1-A xenograft tumors were immunohistochemically stained with Ki67 antibody and Ki67-positive cells were quantitated. **D** Schematic representation of ex vivo culture model. **E** PDEx tissues were treated with EC359 for 72 h and the proliferation was determined using Ki67 immunostaining. Representative Ki67 staining from one PDEx treated with vehicle or EC359 is shown. **F** Ki67 expression in EC explants (*n* = 4) is quantitated. **G** EC PDX (#6564) tumor-bearing mice (*n* = 5) were treated with vehicle or EC359 (5 mg/kg/ip/day/3 times a week). Tumor volumes are shown in the graph. **H** Tumor weights of vehicle and EC359-treated groups are shown. Data are represented as mean ± SE. **p < 0.01, ****p < 0.0001.

To test the efficacy of EC359 in vivo, we have conducted xenograft studies using Ishikawa and HEC-1-A xenograft models. Ishikawa and HEC-1-A cells (2 × 10^6^) were injected subcutaneously (s.c.) into SCID mice. Following the establishment of tumors, mice were randomized and treated for 6 days/week with either 2.5 mg/kg/s.c. of EC359 or vehicle. Compared to the vehicle, EC359 treatment resulted in ~72% and ~74% reduction of tumor volume in Ishikawa and HEC-1-A xenograft models, respectively (Fig. 6A, B). Furthermore, the body weights of the mice in the vehicle and EC359-treated groups did not show significant differences (Supplementary Fig. S1A, B). Further, IHC analysis revealed that EC359-treated HEC-1-A xenograft tumors had decreased number of Ki67-positive cells compared to vehicle-treated tumors (Fig. 6C).

### EC359 reduced the proliferation of primary patient-derived EC explants, organoids, and xenograft tumors

We recently adapted primary human EC explant culture using previously published protocols for culture of tumor tissues [28, 29]. The ex vivo culture model maintains the native tissue architecture and critical cell-to-cell signaling of the tumor microenvironment (TME), and better recapitulates the structural complexity and individual heterogeneity of human cancer in a laboratory setting [25]. Briefly, surgically extirpated de-identified primary EC tissues were cut into small pieces and placed on gelatin sponges soaked in the culture medium and grown for a short term in the presence of vehicle or EC359 (Fig. 6D). Treatment of EC explants with EC359 substantially decreased their proliferation (Ki67 positivity) compared to vehicle-treated tumors (Fig. 6E, F). Next, we tested the effect of EC359 on the growth of organoids established from primary EC tissues. Cell viability analysis of organoids indicated that EC359 treatment significantly reduced their viability compared to vehicle treatment (Supplementary Fig. S2). Importantly, EC359 treatment significantly reduced PDX tumor growth compared to the vehicle-treated control group (Fig. 6G, H). The bodyweight of the mice in the vehicle and EC359 treatment groups did not show significant difference **(**Supplementary Fig. S1C). Collectively, these results demonstrate that EC359 has the potential to reduce the growth of EC in PDEx, PDO, and PDX models.

## Discussion

LIF and LIFR are overexpressed in multiple solid tumors and LIF/LIFR signaling promotes tumor growth, metastasis, and therapy resistance [10, 30]. Tumors exhibit upregulated LIF/LIFR-JAK-STAT3 signaling via autocrine and paracrine mechanisms [15, 31, 32]. LIF signaling promotes crosstalk between tumor cells and fibroblasts and mediates pro-invasive activation of stromal fibroblasts [33]. However, the significance and therapeutic potential of LIF/LIFR in EC is elusive. In this study, we investigated the role of the LIF/LIFR axis in regulating the EC growth, survival, and progression.

Our results showed that EC cell lines and tumors express LIF and LIFR and exhibit autocrine activation of LIF/LIFR downstream signaling, including activation of STAT3. Using, CRISPR KO of LIFR, we provided genetic evidence that LIFR plays a critical role in EC progression in vivo. Blockage of LIF/LIFR axis using LIFR inhibitor EC359 decreased EC cell viability and promoted apoptosis. Mechanistic studies using western blot, RT-qPCR, and reporter gene assays confirmed that EC359 treatment contributed to a significant reduction of LIF/LIFR downstream signaling. Using, EC xenografts, and PDX models, we demonstrated the in vivo efficacy of EC359 in treating EC.

LIF is an established E2-responsive gene in the uterus [22]. LIF is a key paracrine factor from stromal cells acting on cancer cells; and LIF blockade or genetic LIFR deletion slows tumor progression, and augments the efficacy of chemotherapy to prolong survival of pancreatic ductal adenocarcinoma [PDAC] [34]. Blockade of LIF by neutralizing antibodies is shown as an attractive approach in improving cancer therapeutic outcomes [35]. In agreement with these studies, our results provided the evidence that knockout of LIFR compromised tumor progression in EC. We recently designed a small chemical molecule that functions as a high-affinity LIFR inhibitor, EC359. In our study, we identified that inhibition of LIFR using EC359 reduced the growth of established and primary EC cells with high potency and promoted apoptosis.

Our results using EC TMAs demonstrated that EEC have higher expression of LIF and LIFR compared to controls. Together, these results strongly suggest that LIF/LIFR signaling in EC may be clinically actionable and LIFR inhibitor EC359 may be useful in inhibiting LIF/LIFR autocrine loop in EC progression. Preclinical xenograft, PDEx, and PDX studies conducted in this study demonstrated that EC359 is potent in reducing EC tumor growth and the therapy is well tolerated. A potential limitation of our studies is that the EC TMAs consist of a small cohort of samples for EC subtypes. Therefore, future studies are needed using larger number of EC samples with different subtypes to clearly demonstrate whether LIF/LIFR signaling only play a role in endometrioid subtype or is equally important in other subtypes of EC.

Obesity increases the local concentration of E2 levels which directly promote EEC proliferation. LIF is an established E2-responsive gene in the uterus [22]. Tumors often exhibit upregulated LIF/LIFR-JAK-STAT3 signaling via autocrine and paracrine mechanisms [15, 31, 32]. LIF signaling promotes crosstalk between tumor cells and fibroblasts, and mediate pro-invasive activation of stromal fibroblasts [33]. Obese people have unusually high levels of leptin; and leptin increases p-STAT3, LIF, and LIFR levels in cultured human endometrial cells [21]. These emerging studies suggest the critical role of LIF/LIFR signaling in obesity-induced EC progression. Future studies that examine the efficacy of LIFR inhibitor on obesity-associated EC are clearly needed and are beyond the scope of the present study.

LIF is a key regulator of CSCs [10], plays a role in stem-cell maintenance [12, 13, 36], regulates self-renewal and pluripotency [12], and is associated with chemoresistance [14, 15]. Subsequently, LIF/LIFR activates multiple signaling pathways including JAK/STAT3 as immediate effectors, and concurrent MAPK, AKT, and mTOR activation further downstream, all of which are implicated in EC progression [37]. LIFR inhibitor EC359 treatment reduced the activation of STAT3 signaling and its downstream effectors in EC cells and CSCs. Our results indicate EC359 mediates anti-tumor activities both by decreasing proliferation and by increasing apoptosis. Further, LIFR inhibition using EC359 significantly reduced the expression of stemness markers such as SOX2, OCT4, NANOG, c-MYC, and Axin2 in CSCs confirming that EC359 also affects the stemness in addition to proliferation and apoptosis.

In summary, this study tested the novel concept of LIF signaling via LIFR plays a critical role in EC progression. Our data suggest that a LIF/LIFR autocrine loop exists in EC cells and tumors. Using KO of LIFR we provided evidence that the LIFR contributes to EC progression. Further, LIFR inhibitor EC359 blocks LIF/LIFR signaling in EC cells and reduced the cell viability of EC cells both in vitro and in vivo. Our results support LIFR inhibitor EC359 as a novel targeted therapy for EC to target LIF/LIFR oncogenic signaling.

## Materials and methods

### Cell culture and reagents

Human EC cell lines HEC-1-A, AN3 CA, RL95-2 were purchased from the American Type Culture Collection (ATCC, Manassas, VA, USA) and were maintained as per ATCC guidelines and used from early passages. Ishikawa cells were purchased from Sigma (Millipore Sigma, St. Louis, MO, USA). All model cells utilized were free of mycoplasma contamination and were confirmed by using the Mycoplasma PCR Detection Kit purchased from Sigma. Short-tandem repeat polymorphism analysis (STR) of the cells was performed to confirm identity. All these four cell lines belong to endometrioid endometrial cancer (EEC) subtype of EC. The ALDEFLUOR assay kit and MammoCult Human Medium kit were obtained from StemCell Technologies (Cambridge, MA, USA). LIF, LIFR, and Axin2 antibodies were purchased from Santa Cruz Biotechnology (Dallas, TX, USA). The p-Akt(S473), Akt, p-mTOR(S2448), mTOR, pS6(S235/236), S6, p-STAT3(Y705), and STAT3 antibodies were obtained from Cell Signaling Technology (Beverly, MA, USA). The Ki67 antibody was purchased from Abcam (Cambridge, MA, USA). β-actin and all secondary antibodies were purchased from Sigma. LIFR-KO model cells were generated using Genescript (Piscataway, NJ, USA) CRISPR gRNA Constructs (Genescript-s64729-LIFR CRISPR guide RNA 1; Genescript-s64731-LIFR CRISPR guide RNA 2) and transfecting them into Cas9 stably expressing Ishikawa and AN3 CA cells followed by puromycin selection. EC359 was developed by Evestra Inc. (San Antonio, TX, USA) and the detailed synthetic protocol has been described in the patent WO 2016/154203 A1.

### Primary EC cells and EC tissue microarray

Primary EC cells were established from patient-derived EC tissue specimens (Supplementary Table S2) using a University of Texas Health San Antonio (UTHSA) Institutional Review Board approved protocol. These specimens were de-identified; both the PI and research staff did not have access to clinical linkers or codes. All cell lines were maintained in a humidified chamber with 5% CO_2_ at 37 °C. All the methods involving human tissues were conducted in accordance with the declaration of Helsinki and the standards defined by the UTHSA Institutional Review Board.

### Spheroid formation, extreme limiting dilution assays (ELDA), and flow cytometry

Cancer stem cells (CSCs) from Ishikawa cells were sorted using an established stem-cell marker ALDH using ALDEFLUOR kit [38, 39]. For CSCs spheroid formation assays, single-cell suspensions of CSCs were seeded in 24-well ultra-low attachment plates (100 cells/well) in triplicate and treated with vehicle or EC359 (100 and 1000 nM) for 7 days and the newly formed spheres were counted. The effect of EC359 on the self-renewal of CSCs was determined by ELDA. Briefly, CSCs were seeded in decreasing numbers (100, 50, 20, 10, 5, and 1 cells/well) in 96-well ultra-low attachment plates and treated with vehicle or EC359. After 14 days, the number of wells containing spheres per each plating density was recorded and stem-cell frequency between control and treatment groups was calculated using ELDA software (http://bioinf.wehi.edu.au/software/elda/). To determine the effect of EC359 on percent of Axin2-positive CSCs, HEC-1-A and Ishikawa cells were treated with EC359 for 48 h and harvested cells were fixed in 4% paraformaldehyde followed by incubation with 0.1% Triton X-100. Cells were then stained with FITC-conjugated Axin2 antibody as per the manufacturer’s protocol and the percent of the positive cell population was analyzed using flow cytometry.

### Cell viability, clonogenic, and apoptosis assays

The effect of EC359 on cell viability of EC cells was assessed by using MTT cell viability assay as previously described [25]. To test the effect of EC359 on the viability of CSCs, CellTiter-Glo assays were performed (Promega, Madison, WI, USA). For clonogenic survival assays, EC cells were seeded in triplicate in 6 well plates (500 cells/well), after overnight incubation cells were treated with vehicle or EC359 for 5 days and after 2 weeks, colonies that contained ≥50 cells were counted and used in the analysis. The effect of EC359 on apoptosis was analyzed by using the Annexin V/PI kit as per the manufacturer’s instructions (BioLegend, San Diego, CA, USA). Briefly, EC cells were treated with either vehicle or EC359 for 48 h and cells were harvested at a density of 1 × 10^6^ cells/mL in Annexin V binding buffer. Following this step, 100 µL of cell suspension was incubated with Annexin V FITC and propidium iodide (PI) for 15 min at room temperature in the dark. Lastly, 400 µL of Annexin V binding buffer was added to each sample and stained cells were analyzed using flow cytometry.

### Western blotting and RT-qPCR

Whole cell lysates were prepared by using RIPA buffer, and western blot analysis was done using antibodies as previously described [25]. Reverse transcription (RT) reactions were performed by using SuperScript III First Strand kit (Invitrogen, Carlsbad, CA, USA), according to manufacturer’s protocol. Real-time PCR was done using PowerUp SYBR Green master mix (Applied Biosystems, Foster City, CA, USA) on a CFX96 Real-Time PCR system. Primer sequences are included in Supplementary Table S1.

### Reporter gene assays

For STAT3-luc assays, EC cells were stably transduced with STAT3-firefly luciferase reporter lentiviral particles purchased from Cellomic Technology (Helethrone, MD, USA). STAT3-luc reporter expressing cells were serum-starved overnight and treated with EC359 for 24 h. Cells were lysed in a passive lysis buffer, and the luciferase activity was measured by the luciferase assay system (Promega, Madison, WI, USA) using a luminometer.

### Tissue microarray and immunohistochemistry (IHC)

Endometrial carcinoma (EC) tumor microarray (TMA-UT801a) was purchased from US Biomax, Inc. (Rockville, MD, USA). EC tissue microarray contains 24 cases of endometrioid adenocarcinoma (EEC), 4 cases of adenosquamous carcinoma, 6 cases of metastatic endometrioid carcinoma, 20 cases of endometrial hyperplasia, and 15 normal endometrium tissues. Normal tissues are from both post-menopausal and pre-menopausal women. IHC analysis was performed as previously described [40]. Tissue microarrays were probed with both the LIF and LIFR antibodies. Xenograft tumor sections were incubated with Ki67 primary antibody overnight at 4 °C followed by secondary antibody incubation for 45 min at room temperature. Immunoreactivity was visualized by using the DAB substrate and counterstained with hematoxylin (Vector Lab, Burlingame, CA, USA).

### In vivo xenograft models

All animal experiments were performed after obtaining UT Health San Antonio IACUC approval, and all the methods were carried out in accordance with IACUC guidelines. Ishikawa-Vector, and Ishikawa-LIFR-KO model cells (2 × 10^6^) were mixed with an equal volume of growth factor reduced Matrigel and injected subcutaneously into 8-week-old female SCID mice (*n* = 8). For EC359 xenograft studies, Ishikawa (*n* = 4, pilot study) and HEC-1-A (*n* = 3, pilot study) cells (2 × 10^6^) were mixed with equal volume of growth factor reduced matrigel and implanted subcutaneously into SCID mice. After tumor establishment, and achievement of measurable size, mice were randomized into control and treatment groups. The control group received vehicle (0.3% Hydroxypropyl cellulose) and the treatment group received EC359 (2.5 mg/kg/day). All mice were monitored daily for adverse toxic effects. Tumor growth was measured with a caliper at 3–4-days intervals, and volume was calculated using a modified ellipsoidal formula: tumor volume = 1/2(*L* × *W*^2^), where *L* is the longitudinal diameter and *W* is the transverse diameter. At the end of the experiment, mice were euthanized, and tumors were excised, weighed, and processed for histological studies.

### Patient-derived explant (PDEx), organoid (PDO), and xenograft (PDX) studies

For patient-derived explant (PDEx) studies, excised tissue samples were processed, and cultured ex vivo as previously described [25]. De-identified EC tissues were obtained from the UTHSA Ob/Gyn after IRB approval. Briefly, tumor samples were excised and cut into small pieces, and incubated on gelatin sponges for 24 h in a culture medium containing 10% FBS. Tissues were treated with vehicle or EC359 in culture medium for 72 h and fixed in 10% buffered formalin at 4 °C overnight and subsequently processed into paraffin blocks. Sections were then processed for Ki67 immunohistochemical analysis. Patient-derived organoids (PDO) established from de-identified EC tumor tissues were cultured as described in the ATCC culture guides (https://www.atcc.org/en/Guides.aspx). For cell viability assays, PDO’s were harvested from Matrigel and dissociated into single cells using Dispase II (supplemented with ROCKi) and mechanical dispersion. The cell suspension was resuspended in 70% Matrigel and 5 × 10^3^ cells/10 μL drop were seeded per well of a 96-well plate. Culture medium was added, and organoids were allowed to grow for 2 weeks. A concentration dilution series of EC359 or vehicle (DMSO) control was applied to the organoid cultures (in triplicate). Cell viability was assayed after 7 days of treatment using the Promega® CellTiter-Glo® 3D-Superior Cell Viability Assay reagent following the manufacturer’s instructions (Promega, Madison, WI, USA). The intensity of luminescence was measured using a GloMax® Discover System (Promega, Madison, WI, USA). For patient-derived xenograft (PDX) studies, endometrioid PDX tumor (#6564) grown in NSG mice, cut into small pieces (2 mm^3^) and these pieces were reimplanted into the flanks of NSG mice. The mice were then randomized when the tumor volume is ~150–200 mm^3^ into control or treatment groups (*n* = 5 tumors per group). The control group received vehicle (0.3% hydroxy cellulose) and the treatment group received EC359 (5 mg/kg/ip/day) 3 days per week.

### Statistical analyses

Statistical differences between groups were analyzed with unpaired Student’s *t*-test or one-way ANOVA using GraphPad Prism 8 software. All the data represented in plots are shown as means ± SE. A value of *p* < 0.05 was considered statistically significant. For animal studies, a sample size of PDX tumors/treatment was derived using effect information from previous studies, and calculations were based on a model of unpaired data power = 0.8; *p* < 0.05.

## Supplementary information

Supplementary table and figure legends

Supplementary table S1

Supplementary table S2

Supplementary figure S1

Supplementary figure S2

Author contribution
