# Supplementary material for: LIF/LIFR oncogenic signaling is a novel therapeutic target in endometrial cancer
Source: Cell Death Discov. 2021 Aug 16;7:216. doi: 10.1038/s41420-021-00603-z (PMC8367961; doi:10.1038/s41420-021-00603-z)
Supplement: Supplementary file 1 — Supplementary table and figure legends [file 41420_2021_603_MOESM1_ESM.docx]

**Supplementary Table S1**: List of primers used for RT-qPCR analysis

**Supplementary Table S2**: List of primary EC cells used for the study

**Supplementary Figure S1**: Effect of EC359 on body weights of Ishikawa (A), HEC-1-A (B) xenograft and 6564-PDX (C) models.

**Supplementary Figure S2**: A, representative images of PDOs treated with either vehicle or EC359 were shown. B, PDOs treated with either vehicle or EC359 and cell viability was measured after 7 days of treatment
