# Supplementary figures and images for: LIF/LIFR oncogenic signaling is a novel therapeutic target in endometrial cancer
Source: Cell Death Discov. 2021 Aug 16;7:216. doi: 10.1038/s41420-021-00603-z (PMC8367961; doi:10.1038/s41420-021-00603-z)

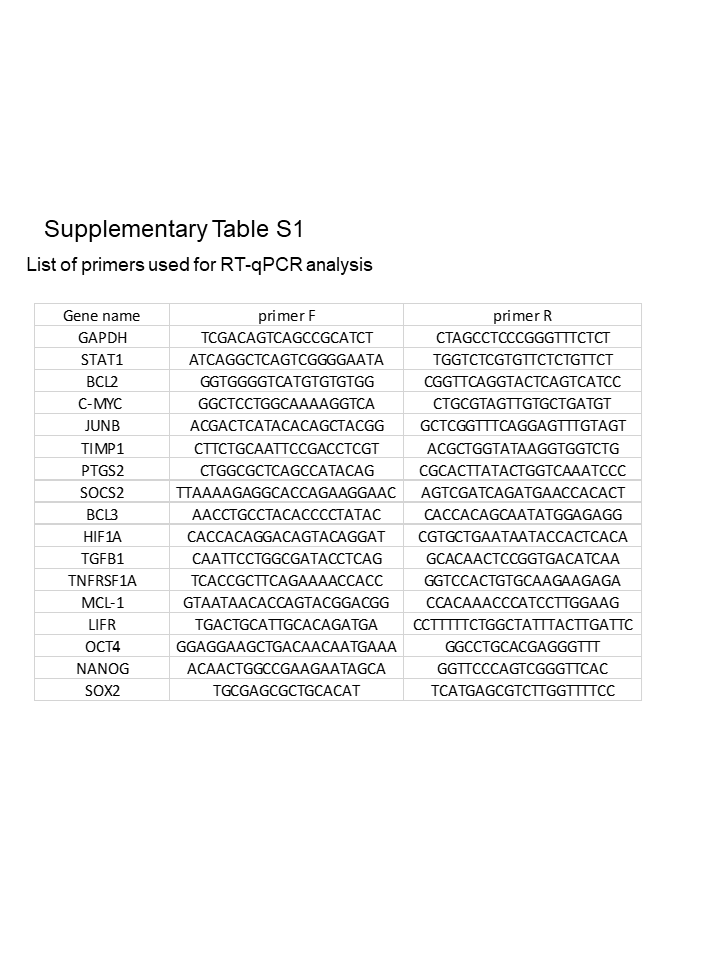

Supplement: Supplementary file 2 — Supplementary table S1 [file 41420_2021_603_MOESM2_ESM.tif]

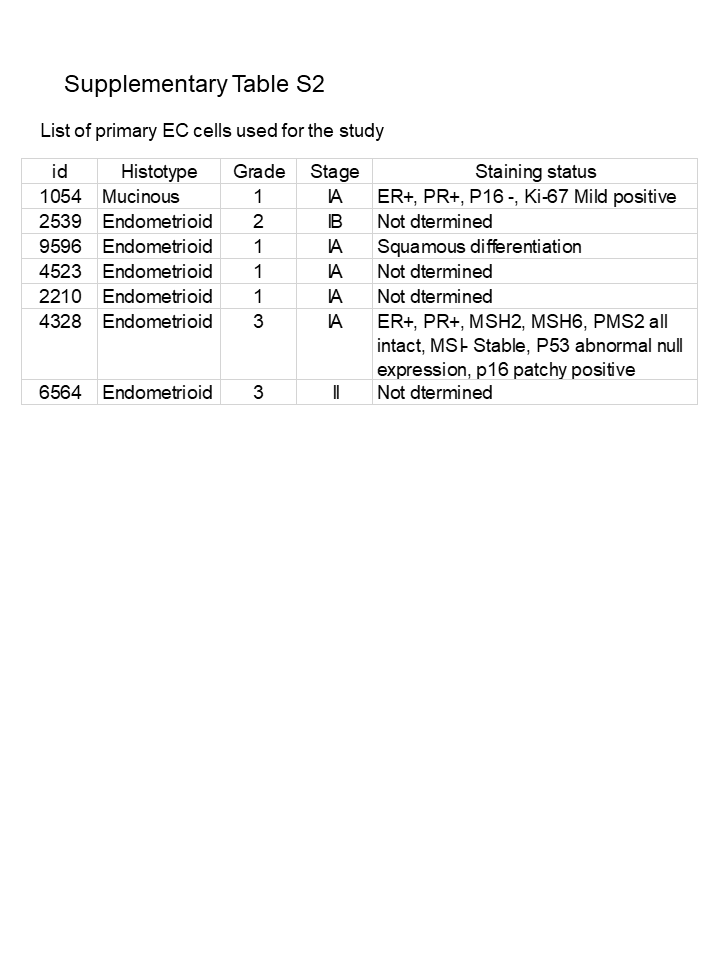

Supplement: Supplementary file 3 — Supplementary table S2 [file 41420_2021_603_MOESM3_ESM.tif]

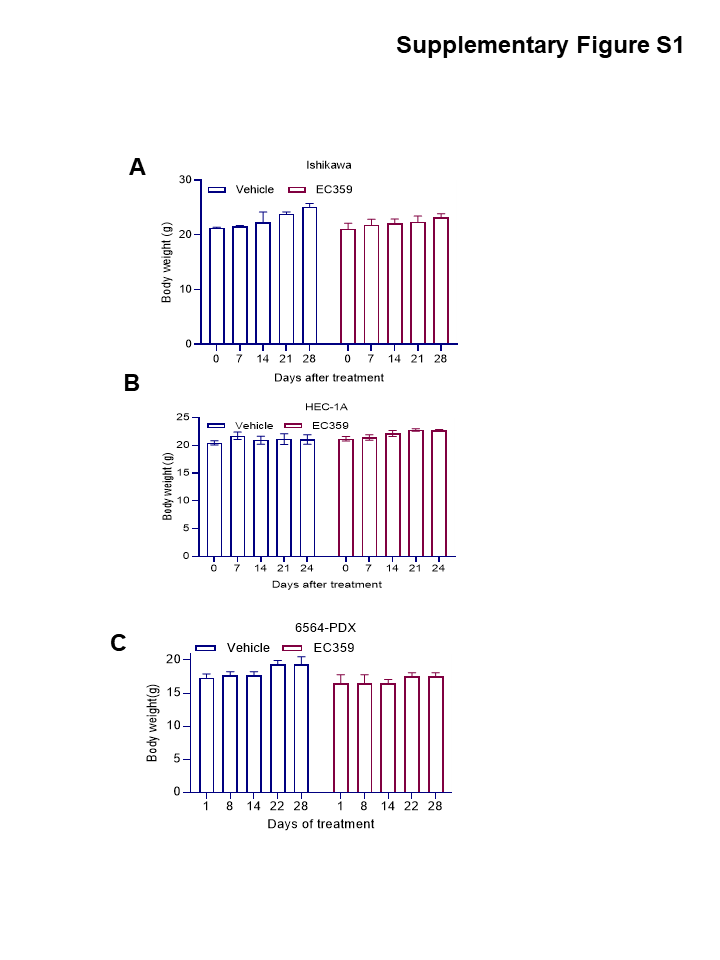

Supplement: Supplementary file 4 — Supplementary figure S1 [file 41420_2021_603_MOESM4_ESM.tif]

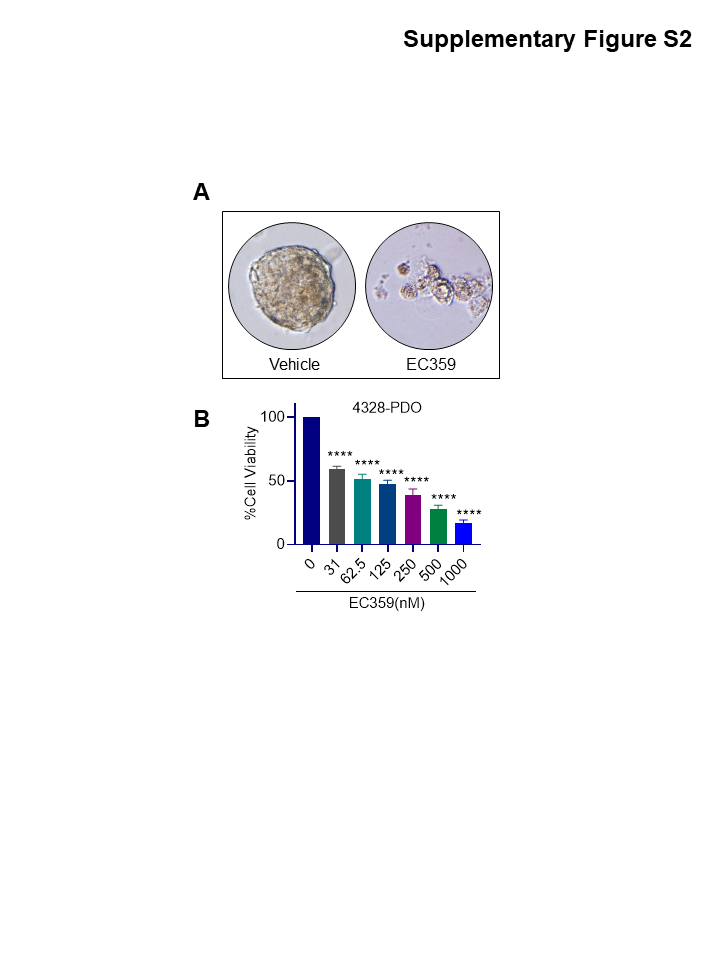

Supplement: Supplementary file 5 — Supplementary figure S2 [file 41420_2021_603_MOESM5_ESM.tif]
